# Changes in prefrontal hemodynamics and mood states during screen use: a functional near-infrared spectroscopy study

**DOI:** 10.1038/s41598-025-09360-w

**Published:** 2025-08-01

**Authors:** Daniel Fehring, Alexandra Gaillard, Emiliano Mazzoli, Susan Rossell, Paddy Dempsey, Michael Wheeler, Neville Owen, David W. Dunstan, Mats Hallgren

**Affiliations:** 1 Turner Institute for Brain and Mental Health, School of Psychological Sciences, Monash University, Melbourne, Australia; 2 Addiction and Clinical Neurosciences Laboratory, Department of Health Sciences and Biostatistics, Swinburne University, Melbourne, Australia; 3 School of Health and Social Development, Institute for Physical Activity and Nutrition, Deakin University, Burwood, Australia; 4 School of Health Sciences, Swinburne University of Technology, Melbourne, Australia; 5 Baker-Deakin Department of Lifestyle and Diabetes, Institute for Physical Activity and Nutrition, Deakin University, Burwood, Australia; 6 Diabetes Research Centre, Leicester General Hospital, University of Leicester, Leicester, UK; 7 MRC Epidemiology Unit, Institute of Metabolic Science, University of Cambridge, Cambridge Biomedical Campus, Cambridge, UK; 8 Baker Heart and Diabetes Institute, Melbourne, Australia; 9 Department of Global Public Health Sciences, Karolinska Institute, Solnavägen 1E, 113 64 Stockholm, Sweden

**Keywords:** Affect, Mood, Sedentary, Cognition, Mentally active, fNIRS, Neuroscience, Psychology

## Abstract

Screen use has been associated with poor cognitive and mental health, yet few studies have examined its effects on brain activity. Our aims were to describe changes in brain activity and mood states following brief exposure to screen-based content; assess the feasibility of using functional near-infrared spectroscopy (fNIRS) to measure these effects; and gather preliminary data to inform future investigations. Twenty-seven young people (age = 21.5 ± 2.8 years; range = 18–25) completed six consecutive 3-min screen conditions in a psuedorandomized cross over design. All screen exposures were presented on an iPhone 12-Max while sitting. Hemodynamic changes in the dorsolateral prefrontal cortex (dlPFC) were measured continuously using fNIRS (Portalite Mk II). Changes in mood states (energy, tension, focus, happiness) were assessed before and after each condition. Condition exposure altered the hemodynamic response in the dlPFC, where oxygenated hemoglobin (HbO) increased more compared to baseline after exposure to social media (largest increase), gaming, and TV-viewing (smallest increase), respectively. Deoxygenated hemoglobin (HbR) and total hemoglobin (HbT) increased more following exposure to gaming (largest increase), social media, and TV-viewing (smallest increase), respectively. Both TV-viewing and gaming were associated with increased focus relative to baseline, whereas social media use was associated with decreased focus. Findings indicate that even short durations of screen use have measurable effects on brain regions involved in cognitive control, emotion, and social decision making. These effects are nuanced and context dependent, rather than universally beneficial or detrimental. fNIRS is a feasible method for measuring these effects.

**Supplementary Information:**

The online version contains supplementary material available at 10.1038/s41598-025-09360-w.

## Introduction

Mood disorders are a major contributor to the global disease burden among young people, with evidence suggesting their prevalence is increasing worldwide^1^. While the causes are multifaceted^2^, rapid increases in screen use (i.e., computers, tablets, and mobile phones) may be a contributing factor^3,4^.

More than ninety percent of young people in Australia own a smart phone^5^; most use screens during leisure time for ≥ 3 h per day, and usage typically peaks during adolescence and early adulthood^6^.

Research suggests that excessive screen time in young people can negatively impact cognitive abilities, including attention and executive functioning^7^. Some studies indicate thinning of the brain’s cortex in areas linked to emotion regulation and reasoning^8^.

Mood disorders are typically preceded by changes in the frequency and/or intensity of negative mood states such as sadness, tension, and irritability^9^. These shorter more reactive emotional states typically increase during the prodrome phase of depression and anxiety disorders^10,11^. A recent study in *Scientific Reports* also found that greater mood ‘variability’ was detrimentally associated with brain development in adolescents and young adults (aged 11–21 years)^11^. Examining the acute effects of screen use on brain activity and mood states could provide valuable new insights into the immediate effects of these ubiquitous behaviors on brain regions linked to emotion regulation. Some observational studies indicate that social media use is associated with negative mood states, especially among girls^12,13^. Several studies have also linked screen use to sedentary behavior (sitting), reduced physical activity and disrupted sleep, which can detrimentally impact mood, wellbeing, and mental health^4,14,15^.

These relationships are complex, nuanced, and dose dependent. For example, one study showed that ‘moderate’ durations of gaming were beneficially associated with mental health in adolescent boys, partly due to increased social interaction and improvements in self-efficacy^16^. Other studies have linked gaming to increases in depressive symptoms^17^. We have shown that in adults, mentally active screen use involving learning and/or problem solving (e.g., work or school related screen use) may have beneficial associations with mental health, despite low energy expenditure^18–20^. Conversely, mentally passive screen use (e.g., TV-viewing) has consistently been linked to elevated depressive symptoms in adults and adolescents^21^. In a longitudinal study (n = 4599 adolescents aged 14–17 years), we demonstrated that replacing 60 min of TV-viewing or social media use with moderate to vigorous physical activity was associated with a reduction in emotional symptoms, including depression, suggesting that screen use may have negative effects on mental health by displacing healthy behaviors (exercise, social interaction, etc.)^22^. Prolonged sitting has been associated with reduced cerebral blood flow in the prefrontal cortex and with lowered mood states^23^; however, it remains unclear whether these associations are attenuated by cognitive activity. Epidemiological studies of screen use have increased exponentially^24–26^, impacting public health recommendations globally^27^, including in Australia, where proposed legislation may ban social media for children and adolescents under 16 years of age^28^. Despite these shifts, experimental studies aiming to elucidate mechanisms that potentially underlie the effects of screen use remain absent.

Functional near-infrared spectroscopy (fNIRS) is a non-invasive neuroimaging technique that uses near-infrared light to measure changes in oxygenated and deoxygenated haemoglobin concentrations in the prefrontal cortex over time; typically following stimulus exposure in experimental settings^29^. Several studies have used fNIRS to measure haemodynamic changes in the dorsolateral prefrontal cortex (dlPFC)—a brain region centrally involved in cognition and emotion regulation—including among children and adolescents^30,31^. fNIRS is widely used in cognitive research due to its safety, portability, low cost, and minimal participant burden^29^.

We conducted an exploratory study to examine the acute effects of three popular forms of screen use (TV-viewing, social media, and gaming) on mood states and prefrontal hemodynamics in the dlPFC. We included young adults aged 18–25 years; a population that uses screens frequently and that may be vulnerable to the deleterious effects of excessive use^5,6^. Our aims for this cross-over study were to: (1) describe changes in brain activity and mood states following brief exposure to screen-based content; (2) assess the feasibility of using fNIRS to examine these acute effects; and (3) gather preliminary data to inform future investigations using fNIRS and other imaging techniques.

## Method

We conducted a three-arm pseudorandomized cross-over study in August–September 2024. Participants (n = 27) were recruited from a convenience sample of healthy young adults at Swinburne University of Technology (SUT), Melbourne, Australia, and reimbursed with a $20 Giftpay card (n = 17), or with course credits for those enrolled in biomedical courses requiring participation in medical device studies (n = 10).

*Inclusion criteria*: Aged 18–25 years, currently uses social media, and fluent in English.

*Exclusion criteria*: A visual impairment that could not be corrected by wearing glasses, and current use of any psychotropic and/or pain medications. Those who did not actively use (past month) social media (e.g., Facebook and Instagram) were also excluded due to requirements of the study procedure.

*Screening*: Students interested in participating were directed to an online screening survey (Qualtrics) to answer short questions on age, education level, English language proficiency, sensory (vision/hearing) impairments, and social media use. Eligible participants were contacted by telephone to confirm eligibility, receive a detailed explanation of the study procedures, provide informed written consent, and schedule a testing session. The study was approved by Swinburne University of Technology Ethics Committee (20248052-18813) and Deakin University Research Ethics Committee (HEAG-H 25_2024) and complied with relevant guidelines and regulations.

### Procedure and measures

**Fig. 1:**
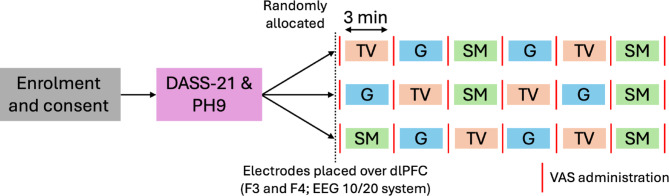
Schematic of study protocol. Participants were enrolled after providing informed consent and completed the Depression, Anxiety, and Stress Scale-21 (DASS-21) and Patient Health Questionnaire-9 (PH9). To avoid sequence effects, they were randomly allocated to one of three screen condition orders (computer generated), each consisting of six 3-min exposures (1-min between conditions): TV (orange), Gaming (G, blue), and Social Media (SM, green). Two exemplars were included per condition to ensure effects reflected broader screen use patterns rather than the specific stimuli. Functional near-infrared spectroscopy (fNIRS) electrodes were placed over the dorsolateral prefrontal cortex (dlPFC) at F3 and F4 (EEG 10/20 system) for continuous brain activity measurement. Visual analogue scales (VAS) were administered after each condition to assess mood changes.

All testing sessions were conducted at Swinburne University of Technology in a noise-attenuated research space. Participants were asked to refrain from drinking alcohol at least 24 h before the experiment. Testing sessions were conducted mid-morning and were divided into two parts taking 60 min to complete (Fig. 1).

#### Part A: baseline questionnaires

Participants completed the 21-item Depression Anxiety Stress Scales (DASS-21), a shorter, validated version of the original DASS tool designed to measure the emotional states of depression, anxiety, and stress (7-items for each)^32^. Participants rated each item based on their experiences over the past week. Participants also completed the Patient Health Questionnaire (PHQ-9), a widely validated tool specifically designed to measure the severity of depressive symptoms^33^. The PHQ-9 consists of 9 self-report items (Likert scale ranging from 0 [not at all] to 4 [nearly every day]), each targeting core symptoms of depression. Scores of 5–9 indicate the absence of depression or subthreshold/mild depressive symptoms; scores of 10–14 may include people with moderate depression; and scores ≥ 15 usually indicate major depression. These questionnaires were included to describe mental health symptoms among the study participants and potentially conduct exploratory analyses based on diagnostic group (depressed/anxious versus not).

#### Part B: experimental condition

To investigate the acute effects of brief sedentary screen use on mood states and prefrontal cortex activity, participants completed six consecutive conditions presented in a psuedorandomized order (computer generated to minimize order effects) with a one-minute seated break between each condition to minimise potential carry-over effects and allow the hemodynamic response to return to baseline (Fig. 1). These conditions represented three screen-use categories, each with two distinct exemplars to enhance generalizability and ensure that observed effects were not driven by a specific stimulus: (1) TV-viewing (*Friends*, a comedy, and a David Attenborough nature documentary), (2) gaming (*Tetris* and *Angry Birds*), and (3) social media use. This design allowed us to assess the broader impact of each screen-use category rather than effects specific to a single program, game, or platform. All conditions were presented on mobile devices: TV-viewing and gaming conditions were displayed on an iPhone 12 Max, while social media was accessed on the participants’ mobile phones on the two platforms they use most frequently used (e.g., Facebook, Instagram, TikTok). This approach ensured that their interactions remained natural and reflective of their typical social media use, allowing for a more personalized and ecologically valid experience. Tetris and Angry Birds are freely available phone games that require active cognitive engagement and problem solving/strategy and have been used in related studies of cognition^34,35^. As the hemodynamic response to audio-visual stimuli occurs rapidly^36^, each exposure lasted for 3 min, resulting in an 18-min total screen exposure (six conditions × 3-min each). For fNIRS analysis, a 2-s baseline immediately preceding each condition and the first 60 s of screen exposure were analysed to capture early hemodynamic changes following stimulus onset. To assess participants’ current mood state, a Visual Analog Scale (VAS) was administered at baseline, as well as immediately before and after exposure to each condition. The four mood states assessed were: Energy (0—*Very Fatigued* to 10—*Very Energetic*), Tension (0—*Absolutely No Tension* to 10—*Very Tense*), Focus (0—*Very Distracted* to 10—*Very Focused*), and Mood (0—*Very Sad* to 10—*Very Happy*). The VAS is a psychometric response scale often used in short duration experimental studies to assess the immediate intensity of subjective mood states. These four mood states were also chosen based on their face validity (relevance to screen use), their association with mood disorders^37^, and brain health among adolescents and young adults^11^.

### Brain activity

**Fig. 2:**
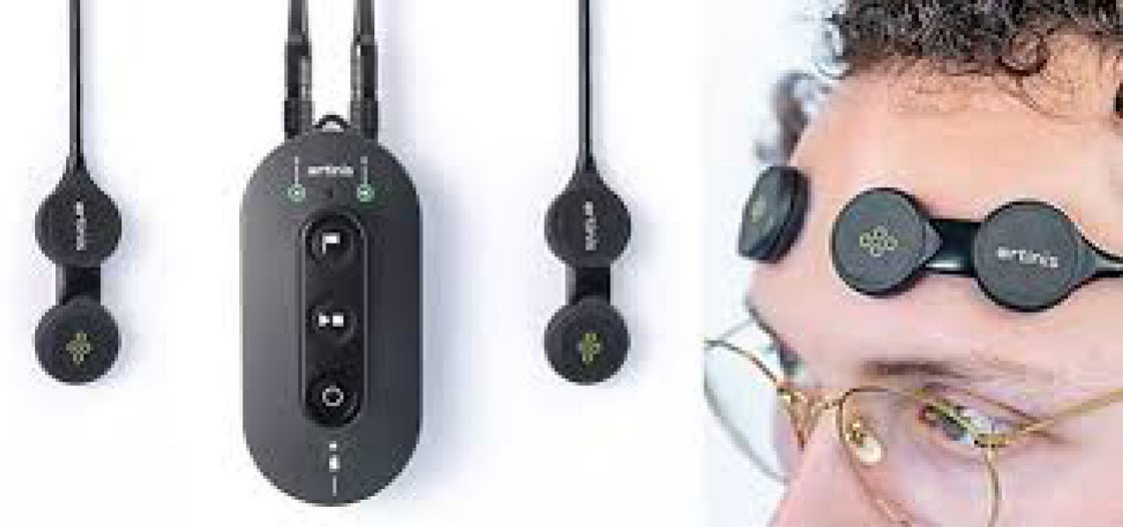
Placement and recording of brain activity (with permission from Artinis Medical Solutions).

During exposure to each screen use condition, changes in cerebral oxygenation in the dorsolateral prefrontal cortex (dlPFC: electrodes over the left and right dlPFC, F3 and F4 EEG 10–20 system [Fig. 2]); was measured using a portable functional near-infrared spectroscopy (fNIRS) device (Portalite MKII, Artinis Medical SystemsTM, The Netherlands). fNIRS is a non-invasive imaging technique that measures cerebral blood flow and oxygenation, providing insights into the neural correlates of cognitive and emotional processing associated with each type of screen use. We focused on activity in the dlPFC as this brain region plays a key role in emotional control (modulation of affective processing), working memory, goal-driven attention, planning, and problem-solving^38^. Reduced activity in the left dlPFC has been observed in studies involving depression, reinforcing its role in emotional regulation^38^. The fNIRS device contained two sensors, each with two photodiode receivers and three transmitters at distances of 29, 35, and 41 mm from the receiver. Each transmitter operated at two wavelengths (760 nm and 850 nm), allowing for measurement of the changes in oxygenated hemoglobin (HbO), deoxygenated hemoglobin (HbR), and total hemoglobin (HbT) concentration, with a sampling frequency of 100 Hz.

### fNIRS data preprocessing

Hemoglobin density values were recorded by the NIRS device sent to a laptop via Bluetooth, and stored in Oxysoft (Artinis Medical Systems, Netherlands). Raw values were imported to MATLAB (The MathWorks, Inc., Natick, MA, USA) using the oxysoft2matlab function, and HomER3 software^39^ was used for preprocessing and analysis. Raw optical densities were first converted to optical density (OD). Motion artifacts were automatically identified and corrected using spline interpolation. Additionally, a low-pass filter at 0.50 Hz was used to remove high frequency noise (i.e., cardiac signal), and a high-pass filter at 0.01 Hz was used to remove low-frequency noise (i.e., data drift). After this, optical density rates were converted to hemoglobin concentration for HbO, deoxygenated hemoglobin (HbR), and total hemoglobin (HbT = HbO + HbR) through the modified Beer-Lambert law. HRF was estimated using a General Linear Model (GLM) of ordinary least squares^40^ and applied a sequence of consecutive Gaussian function to model the HRF shape^41^. The model was prepared using a HRF time range determined based on the experimental design, that is − 2 s prior and 60 s after the exposure onset. To ensure physiological oscillations that fall within the same frequency band as the hemodynamic response signals (such as Mayer waves) were removed, short separation regression was applied, with data from the most correlated short channel being used^42,43^. Finally, HbO, HbR, and HbT values during exposure to each condition were exported to SPSS (version 29). For each hemoglobin variable (HbO, HbR, HbT), mean values over all channels were calculated for each condition. Data was prepared by creating segments for baseline and exposure for each condition. Baseline was defined as two seconds before exposure onset, and exposure was defined as 60 s after exposure onset. Mean values were calculated for each segment. For baseline correction, the mean fNIRS signal during baseline was subtracted from exposure fNIRS signals for each condition.

### Statistical analyses

Participant characteristics are reported using descriptive statistics (means and standard deviations (SD)). Changes in mood states and brain activity were examined using repeated measures analysis of variance (ANOVA) with effect sizes (partial eta-square, η^2^) and post-hoc contrasts (t-tests and their associated confidence intervals: CIs). Spearman’s rank-order correlations were conducted to examine the relationship between changes in HbO, HbR, and HbT levels, clinical characteristics, and VAS scores across each condition (gaming, social media, and TV-viewing). Data assumptions were checked, including normality (skewness/kurtosis), homogeneity of variance (Levene’s test) and independence (Durbin-Watson test). To control for Type I errors, multiple comparisons were adjusted using the Bonferroni method, setting the significance threshold for Spearman’s rank-order correlations at an adjusted α of 0.017. All analyses were conducted using SPSS (version 29) and R (version 4.4.1).

## Results

### Participant characteristics

Sociodemographic and clinical characteristics of the sample are shown in Table 1. The severity of depressive symptoms, anxiety, and stress was comparable to those reported in Australian studies of university students^44^. DASS-21 scores indicated 4 (14.8%), 4 (14.8%), and 1 (3.7%) of participants displayed indications of depression, anxiety, and stress, respectively (these were insufficient to run sub-group analyses based on depression status).

**Table 1:**
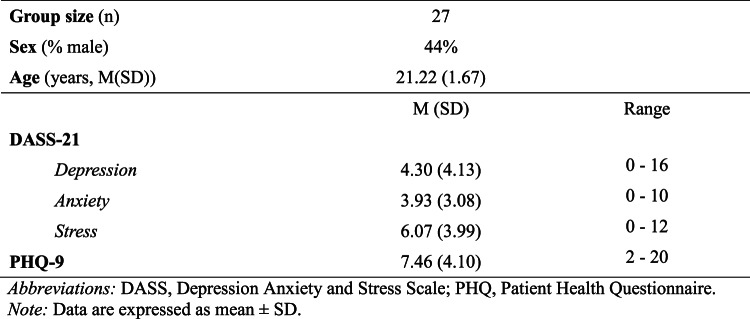
Participant characteristics.

Data are expressed as mean ± SD.

*DASS* depression anxiety and stress scale, *PHQ* patient health questionnaire.

### Effects on brain activity

#### Oxygenated haemoglobin (HbO)

A main effect of condition was observed, F(1.966, 11,795.039) = 468.042, *p* < 0.001, partial η^2^ = 0.072, with HbO values increasing more from baseline after exposure to social media (largest increase) (M = 0.12, SD = 0.18), gaming (M = 0.06, SD = 0.18) and TV-Viewing (smallest increase) (M = 0.02, SD = 0.18), respectively. Post hoc analysis revealed that HbO values were statistically different between gaming and social media (M = − 0.063, 95% CI [− 0.071, − 0.055], *p* < 0.001), gaming and TV-Viewing (M = 0.036, 95% CI [0.029, 0.043], *p* < 0.001), and social media and TV-Viewing (M = 0.099, 95% CI [0.091, 0.107], *p* < 0.001).

#### Deoxygenated haemoglobin (HbR)

A main effect of condition was observed, F(1.990, 11,938.104) = 4473.366, *p* < 0.001, partial η^2^ = 0.427, with HbR values increasing more from baseline after exposure to gaming (largest increase) (M = 0.09, SD = 0.09) and decreasing from baseline after exposure to social media (M =  − 0.02, SD = 0.07), and TV-Viewing (M = − 0.05, SD = 0.07), respectively. Post hoc analysis revealed that HbR values were statistically different between gaming and social media (M = 0.103, 95% CI [0.100, 0.107], *p* < 0.001), gaming and TV-Viewing (M = 0.132, 95% CI [0.128, 0.135], *p* < 0.001), and social media and TV-Viewing (M = 0.029, 95% CI [0.025, 0.032], *p* < 0.001).

#### Total haemoglobin (HbT)

**Fig. 3:**
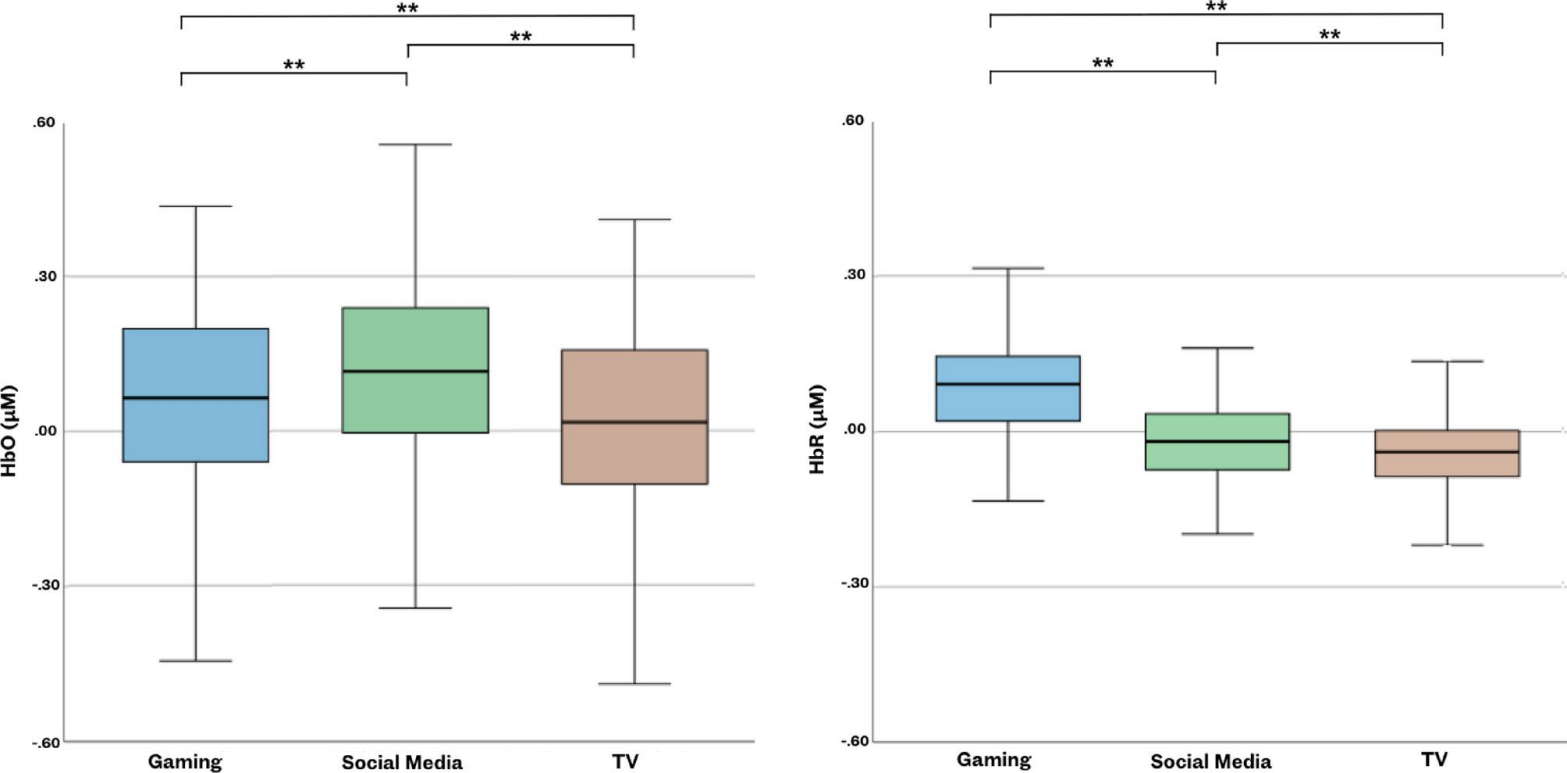
Boxplots of the mean (left) HbO values and (right) HbR values in the dlPFC across all conditions (gaming, social media, TV-Viewing). Asterisk denotes *p* < 0.001*.*

**Fig. 4:**
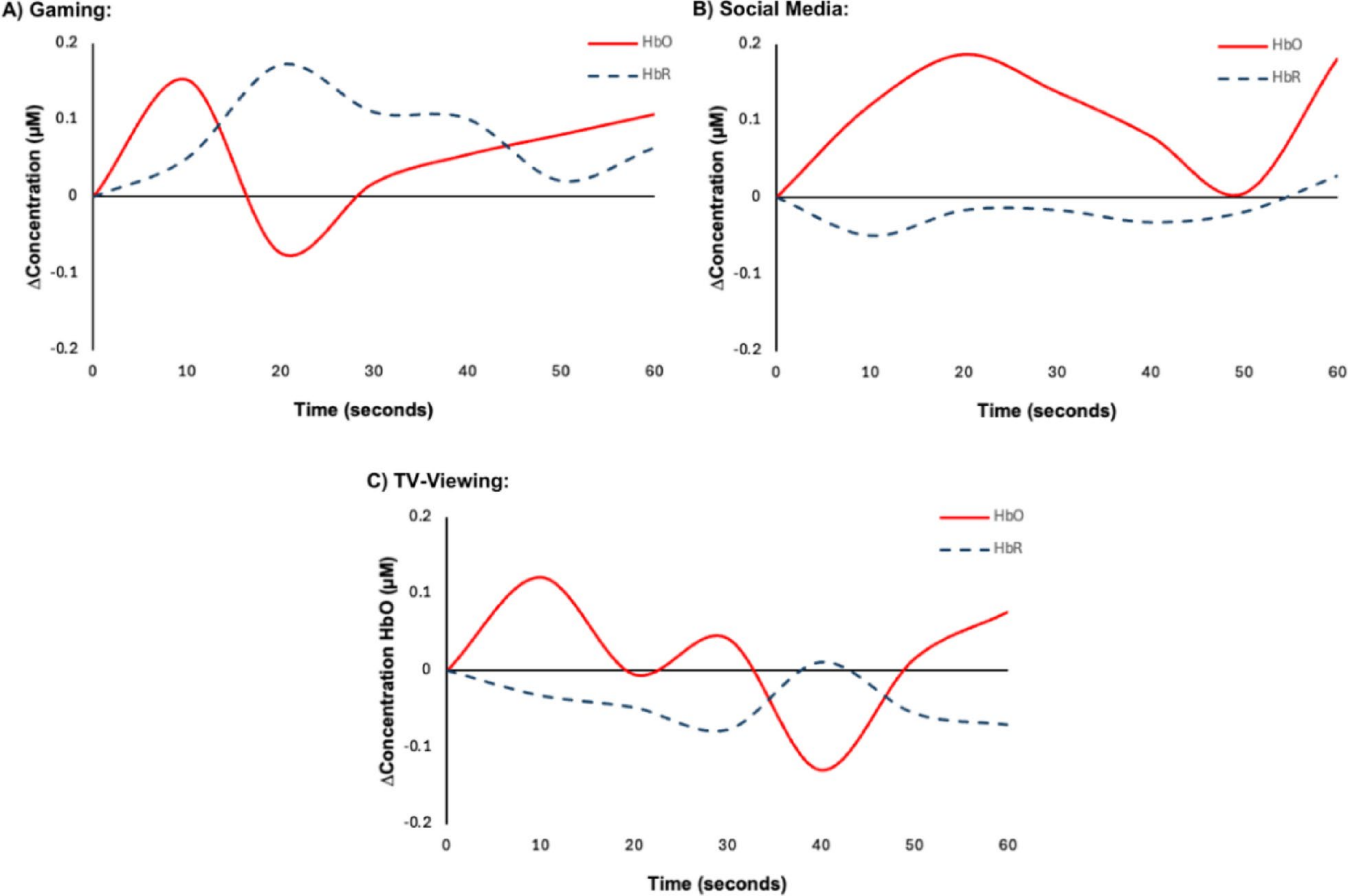
Time plot of change in concentration of oxygenated (HbO) and deoxygenated (HbR) dlPFC acrossall participants during (**A**) gaming, (**B**) social media, and C) TV-Viewing conditions.

A main effect of condition was observed, F(1.963, 11,776.942) = 1189.204, *p* < 0.001, partial η^2^ = 0.165, with HbT values increasing more from baseline after gaming (M = 0.14, SD = 0.19), social media (M = 0.10, SD = 0.21), and TV-Viewing (smallest increase) (M = − 0.03, SD = 0.20), and gaming. Post hoc analysis revealed that HbT values were statistically different between gaming and social media (M = 0.04, 95% CI [0.032, 0.049], *p* < 0.001), gaming and TV-Viewing (M = 0.168, 95% CI [0.160, 0.176], *p* < 0.001), and social media and TV-Viewing (M = 0.128, 95% CI [0.119, 0.137], *p* < 0.001). Results are shown in Figs. 3 and 4, below.

### Effects on mood states

Descriptive data (Mean ± SD) from the VAS for each screen use condition are shown in Table 2. For distraction-focus, a main effect of condition was observed, F(1.990, 43.780) = 6.436, *p* = 0.004, partial η^2^ = 0.226, with focus scores increasing from baseline for TV-viewing and gaming but decreasing for social media. Post-hoc analysis with Bonferroni adjustment revealed that scores were statistically different between gaming and social media (M = 1.326, 95% CI [0.745, 1.907], *p* < 0.001). No statistically significant changes in VAS scores on energy (F(2.260, 47.468) = 2.612, *p* = 0.077, partial η^2^ = 0.111), relaxation-tension (F(1.980, 43.560) = 0.954, *p* = 0.420, partial η^2^ = 0.042), or sadness-happiness (F(3, 69) = 1.647, *p* = 0.187, partial η^2 ^= 0.067) were found between conditions.

**Table 2:** Means and standard deviations from the Visual Analogue Scales (VAS) for each condition.

VAS Scale	Baseline	Gaming	Social media	TV-viewing
Energy	6.59 (1.94)	6.80 (1.94)	6.05 (1.75)	6.32 (1.90)
Distraction-focus	5.87 (1.79)	6.89 (1.31)	5.57 (1.46)	6.28 (1.62)
Relaxation-tension	3.91 (2.43)	4.11 (2.35)	3.54 (2.06)	3.67 (2.26)
Sadness-happiness	6.96 (1.57)	7.13 (1.36)	6.73 (1.37)	6.94 (1.50)

Data are expressed as mean ± SD. Lower scores indicate worse mood states.

*VAS* visual analogue scale.

### Associations between change in brain activity and change in mood states

**Fig. 5:**
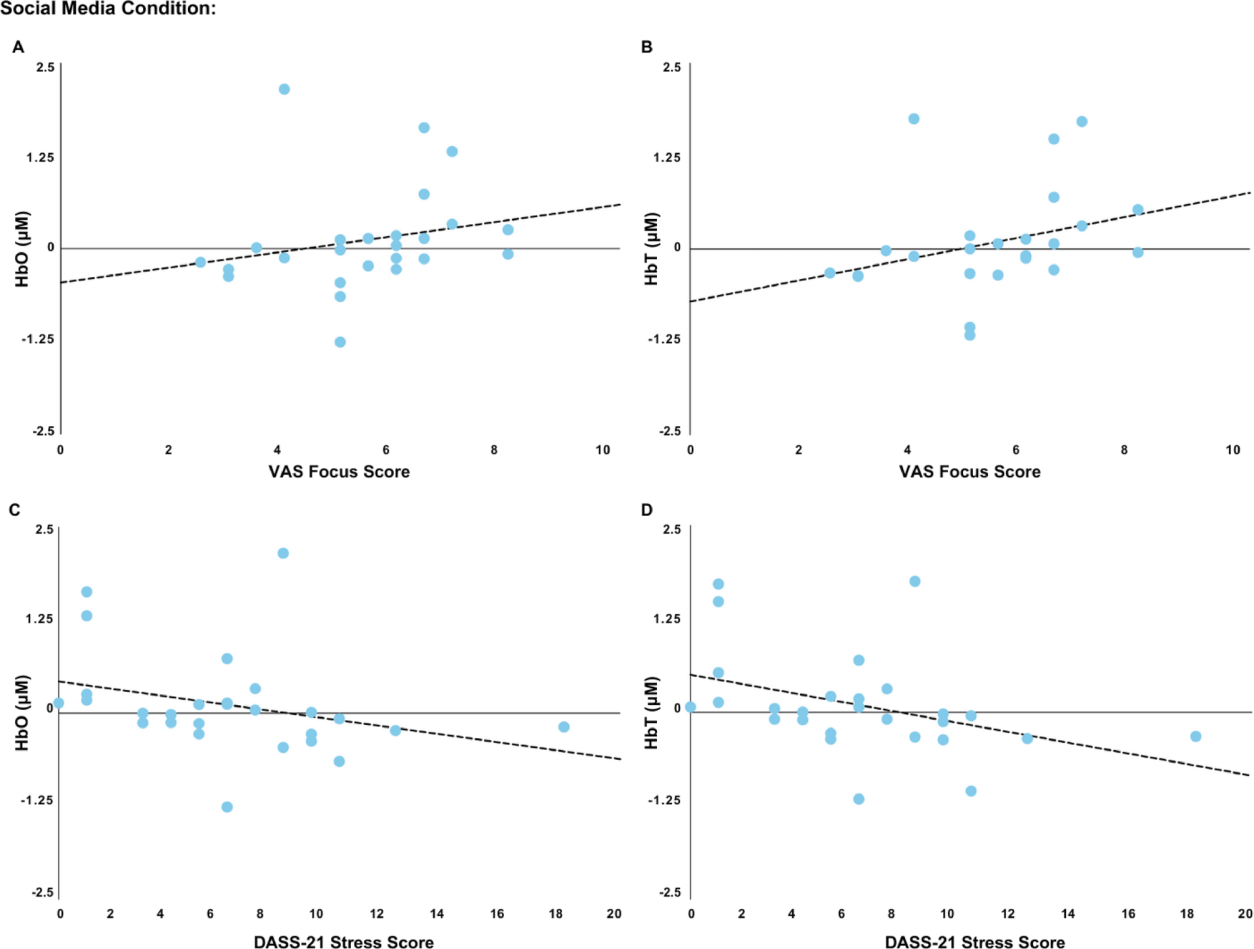
Significant correlations between clinical characteristics, VAS mood states, and oxygenated hemoglobin (HbO) and total hemoglobin (HbT) concentrations in the social media condition. VAS focus score was significantly correlated with (**A**) HbO and (**B**) HbT. DASS-21 stress score was significantly correlated with (**C**) HbO and D) HbT.

To control for Type I errors, multiple comparisons were adjusted using the Bonferroni method, setting the significance threshold for Spearman’s rank-order correlations at an adjusted α of 0.017. Gaming: The correlation between HbR values and VAS tension score during gaming was not significant, rs(25) = 0.435, *p* = 0.030. TV-viewing: The correlation between HbR values and VAS mood score during TV-Viewing, rs(25) = 0.454, *p* = 0.023 was not significant. Social media: There were significant negative correlations between HbO and HbT values and DASS-21 Stress Score, rs(27) = − 0.477 (Fig. 5C), *p* = 0.012 and rs(27) =  − 0.488, *p* = 0.010 (Fig. 5D), respectively, indicating that higher stress at baseline was associated with lower HbO and HbT when viewing social media (Fig. 5). In addition, significant positive correlations were observed between HbO and HbT values and VAS focus score, rs(25) = 0.499, *p* = 0.011 (Fig. 5A) and rs(25) = 0.510, *p* = 0.009 (Fig. 5B), respectively. Supplementary Table 1 shows all correlations between the change in brain activity and the change in mood states.

## Discussion

Our findings confirm for the first time in an experimental setting that different forms of screen use are associated with distinct patterns in dlPFC hemodynamic activity and mood states in young adults, and also demonstrate that fNIRS is a feasible approach to measure these changes (data collection was successful and there were no safety issues or adverse events). Our study adds the key observation that screen use has immediate and measurable hemodynamic effects in the dlPFC; a brain region associated with emotion regulation, attention, memory, and mental health. The findings suggest that even brief exposure to screen-based media can differentially recruit both affective and attentional processes in the dlPFC and reinforce the notion that ‘screen time’ is neither uniformly detrimental nor beneficial but rather nuanced and context dependent.

Exposure to the three screen conditions altered the hemodynamic response of the dlPFC in specific ways. First, compared to baseline, HbO increased after TV-viewing (lowest), gaming, and social media use (highest increase). These changes indicate that cerebral blood flow increased more during the ‘cognitively active’^45^ screen conditions (gaming and social media) compared to TV-viewing. This was similarly observed in HbR, which increased exponentially from TV-viewing (lowest), social media, and gaming (highest increase). This finding is relevant, as previous epidemiological studies have shown detrimental associations between TV-viewing and a range of health outcomes, including pre-mature mortality, mental health and cognition^46,47^. Structural changes in the brain have been reported, including reductions in grey matter^48^. These effects have also been attributed to changes in cardiometabolic risks (e.g., inflammation) linked to sedentary behaviour, and the coinciding consumption of highly processed food^49^. The Coronary Artery Risk Development in Young Adults (CARDIA) study examined the effects of TV-viewing over 20 years in 599 middle-aged adults^50^. After adjustment for covariates, including physical activity, increased TV-viewing time was negatively associated with gray matter volume in the frontal and entorhinal cortex, and total gray matter^50^. These findings suggest that the negative effects of TV-viewing on brain structure and function may, at least in part, be related to its lower cognitive demands compared to more interactive screen-based activities, potentially leading to reduced neural engagement and plasticity over time. However, the causal mechanisms remain unclear; future research could examine (for example) whether the content of TV-viewing influenced dlPFC activity or mediates long-term neurocognitive changes.

The impact of social media, the most prevalent form of screen use, on young people’s mental health has received considerable public and research attention, with some studies linking its use to poor educational outcomes and to attention-related disorders^51–53^. In the current study, both gaming and TV-viewing were associated with an increase in self-reported focus relative to baseline, whereas social media use was associated with a decrease in focus. Subjective focus is a key component of attention and associated with learning outcomes in young adults^54^. A cross-sectional study involving university students aged 17–29 found that 57% were addicted to social media (compulsive use that negatively impacts socio-occupational functioning); 52% reported that social media use had a negative impact on their ability to focus and learn; and 66% were more drawn toward social media than academic learning^55^. A prospective study involving 659 university students in the US found that longer durations of social media use predicted worse academic outcomes^56^; and a recent systematic review of 28 longitudinal studies reported reciprocal associations between social media use and ADHD symptoms in children^53^.

Self-reported focus decreased during social media use, despite an increase in HbO, indicating a potential dissociation between neurophysiological activation (greater oxygenated blood flow) and subjective attentional experience (reduced focus). The underlying mechanism of this incongruence remains unclear; however, we speculate that social media may uniquely disrupt sustained attention by delivering a continuous stream of external stimuli, including notifications, likes, and algorithmically curated content, that reinforce a cycle of instant gratification, potentially impairing the ability to engage in cognitively demanding tasks requiring prolonged focus^57^. Moreover, in the current study, higher stress ratings at baseline were associated with lower HbO and HbT in the dlPFC after social media use, suggesting that participants who experience a higher level of stress received less oxygenated blood flow than those who had reported low stress scores at baseline. Our study was not designed to examine sex differences, however, in a previous longitudinal study (n = 11,341), we found that social media use was detrimentally associated with depressive symptoms among girls but not among boys (aged 11–14 years)^58^. The moderating effect of sex and gender requires further exploration in this context. It is worth noting that the relationship between stress ratings and HbO did not elicit similar relationships with HbR. When interpreted together, this implies that despite greater oxygenated blood flow to the dlPFC in participants experiencing lower levels of stress, there were no corresponding changes in the local metabolic demands of the dlPFC. This is supported by past literature in stress-related disorders where altered neurovascular responses are reported despite demand remaining similar, particularly in the prefrontal cortex^59–61^

Video gaming (gaming) is another screen activity that has received widespread attention, but research findings are inconsistent. In a prospective study, we reported that more frequent gaming was associated with fewer depressive symptoms in boys with low physical activity levels, but these relationships were not seen among girls^58^. A large (n = 2217) cross sectional study of children aged 9–10 years found that gaming was beneficially associated with cognitive functions that involve response inhibition and working memory, but also with higher depression/anxiety symptoms^62^. In contrast, a recent systematic review of randomized controlled trials concluded that video game interventions are an effective treatment for depression (but not anxiety) in adolescents and young adults^63^. In the current study, we found that gaming increased HbO, indicating a greater utilization of oxygen in the dlPFC, however compared to the typical neurovascular coupling response (increased HbO and decreased HbR) observed in both TV-viewing and social media, the increased HbO was accompanied by increased HbR during gaming. Compared to gaming, social media use and TV-viewing are relatively passive screen activities^45^. Thus, the pattern observed during gaming could be indicative of not only to neurophysiological activation (greater oxygenated blood flow), utilization of oxygen in the dlPFC, and the need for elevated oxygen extraction, but sympathetic vasoconstriction, a feature of stress-related physiological responses resulting in the rapid decrease in HbO and further increasing rates of HbR due to reduced HbR washout^64,65^. This is further supported as the increased HbR effect was stronger when tension was rated highly, although this did not survive Bonferroni correction.

Our study demonstrates that fNIRS is a feasible technique for collecting data on hemodynamic activity during screen use, and that it can differentiate the effects of different forms of screen use in the dlPFC. Overall, the findings align with previous observational evidence indicating that the effects of screen use are nuanced and content/context dependent^55,66^. Strengths of the study include the novel application of fNIRS in this context. The pseudorandomized cross-over design enhanced both the internal validity and reliability of the findings by controlling for person-specific factors and permitting a smaller sample size. Given the modest sample, a structured pseudorandomization procedure, rather than full randomization, was necessary to ensure balanced exposure to each condition across participants and sessions. This design is a robust approach for examining the effects of brief interventions and assessment of the impact of each screen-use category. The preliminary findings provide a rational, scientific basis for a larger investigation of these relationships.

Potential limitations include the small sample size and the short exposure time for each condition (3 min), which was long enough to detect hemodynamic changes, but may have limited the capacity to detect changes in mood states. The first minute of screen exposure was used to examine changes, which could partly reflect novelty or familiarity with the stimuli. The use of single item VAS responses is also a potential limitation, although previous studies have shown that these items correlate highly with validated measures of mood^67^. Another limitation is that systemic physiological parameters, such as mean arterial pressure, heart rate, skin conductance, and respiratory rate, were not assessed during the study. These systemic factors could provide important context for interpreting the findings, particularly with respect to the initial hemodynamic response to each condition. These measurements (SPA-fNIRS) should be incorporated into future study designs to better understand the hemodynamic changes associated with screen use^68^. While social media usage was used as a binary inclusion criterion, we did not assess participants’ daily screen time or overall exposure to digital devices. Given emerging evidence suggesting that prolonged screen exposure may influence cerebral hemodynamics, future work should incorporate quantitative measures of screen time and explore its potential interaction with neurophysiological outcomes at the individual level. Qualitative investigations could also provide a deeper understanding of the experiences associated with the content presented within each screen-use category, in particular social media. Examination of longer and more diverse screen exposures (e.g., other forms of social media) is needed, as different content could modify these effects. Finally, the impact of so-called ‘active’ social media use (posting messages, etc.) versus passively scrolling reading should be examined^69^.

In conclusion, our findings underscore the need for a nuanced approach to understanding the effects of screen-use on brain function. We observed that different forms of screen-use were associated with distinct patterns of hemodynamic activity in the dlPFC, and with mood states in young people. The findings indicate that even short durations of screen use have measurable effects on brain regions involved in cognitive control, emotion, and social decision making that are essential for wellbeing.

## Electronic supplementary material

Below is the link to the electronic supplementary material.

Supplementary Material 1

## Data Availability

The datasets generated and/or analysed during the current study are not publicly available as the data contains sensitive material but are available from the corresponding author on reasonable request.
