# Supplementary material for: Changes in prefrontal hemodynamics and mood states during screen use: a functional near-infrared spectroscopy study
Source: Sci Rep. 2025 Aug 1;15:28181. doi: 10.1038/s41598-025-09360-w (PMC12316940; doi:10.1038/s41598-025-09360-w)
Supplement: Supplementary file 1 — Supplementary Material 1 [file 41598_2025_9360_MOESM1_ESM.docx]

**Supplementary Table 1:**

Correlations between changes in brain activity and changes in mood states

|  | **HbO** | | **HbR** | | **HbT** | |
| --- | --- | --- | --- | --- | --- | --- |
|  | ***r*** | ***p*** | ***r*** | ***p*** | ***r*** | ***p*** |
| **Gaming** |  |  |  |  |  |  |
| **DASS-21** |  |  |  |  |  |  |
| *Depression* | .117 | .561 | -.217 | .278 | -.045 | .824 |
| *Anxiety* | .050 | .803 | .079 | .695 | .073 | .717 |
| *Stress* | .309 | .116 | .326 | .097 | .375 | .301 |
| **PHQ-9** | .110 | .609 | -.264 | .212 | -.040 | .853 |
| **VAS** |  |  |  |  |  |  |
| *Energy* | -.063 | .766 | .125 | .553 | .009 | .966 |
| *Focus* | .010 | .961 | .010 | .962 | .012 | .956 |
| *Tension* | .182 | .384 | .435 | .030 | .321 | .118 |
| *Mood* | .249 | .230 | -.148 | .480 | .108 | .608 |
| **Social media** |  |  |  |  |  |  |
| **DASS-21** |  |  |  |  |  |  |
| *Depression* | -.043 | .830 | -.175 | .384 | -.060 | .766 |
| *Anxiety* | -.266 | .180 | -.303 | .125 | -.281 | .155 |
| *Stress* | **-.477** | **.012** | -.365 | .061 | **-.488** | **.010** |
| **PHQ-9** | -.153 | .476 | -.214 | .316 | -.178 | .405 |
| **VAS** |  |  |  |  |  |  |
| *Energy* | .112 | .610 | .235 | .281 | .211 | .335 |
| *Focus* | **.499** | **.011** | .355 | .082 | **.510** | **.009** |
| *Tension* | -.014 | .949 | .053 | .803 | .038 | .856 |
| *Mood* | .146 | .478 | .030 | .883 | .189 | .354 |
| **TV** |  |  |  |  |  |  |
| **DASS-21** |  |  |  |  |  |  |
| *Depression* | -.129 | .520 | -.155 | .440 | -.234 | .241 |
| *Anxiety* | .080 | .692 | -.275 | .165 | -.089 | .660 |
| *Stress* | -.093 | .644 | -.073 | .717 | -.215 | .281 |
| **PHQ-9** | .011 | .958 | -.189 | .377 | -.112 | .603 |
| **VAS** |  |  |  |  |  |  |
| *Energy* | .078 | .709 | .384 | .058 | .075 | .721 |
| *Focus* | -.231 | .277 | .104 | .628 | -.186 | .385 |
| *Tension* | .129 | .530 | -.339 | .090 | .123 | .548 |
| *Mood* | -.261 | .208 | .454 | .023 | -.213 | .308 |

*Abbreviations: HbO,* oxygenated hemoglobin; HbR, deoxygenated hemoglobin; HbT, total hemoglobin; DASS, Depression Anxiety and Stress Scale; PHQ, Patient Health Questionnaire; VAS, Visual Analogue Scale. VAS, Visual Analogue Scale. Spearman *r* correlations shown.
